# Novel *Providencia xianensis* sp. nov.: A multidrug-resistant species identified in clinical infections

**DOI:** 10.1007/s10096-024-04821-y

**Published:** 2024-05-07

**Authors:** Xu Dong, Yanghui Xiang, Peihong Yang, Shanmei Wang, Wenjuan Yan, Youhua Yuan, Shan Zhou, Ke Zhou, Jiayun Liu, Ying Zhang

**Affiliations:** 1 State Key Laboratory for Diagnosis and Treatment of Infectious Diseases, National Clinical Research Center for Infectious Diseases, Collaborative Innovation Center for Diagnosis and Treatment of Infectious Diseases, The First Affiliated Hospital, Zhejiang University School of Medicine, Hangzhou, China; 2 Department of Clinical Laboratory Medicine, Xijing Hospital, Fourth Military Medical University, Xi’an, China; 3 Department of Clinical Microbiology, Henan Provincial People’s Hospital, Zhengzhou University People’s Hospital, Henan University People’s Hospital, Zhengzhou, China; 4 Jinan Microecological Biomedicine Shandong Laboratory, Jinan, China

**Keywords:** Novel species, Multidrug resistance, *Providencia rettgeri*, *Providencia xianensis*

## Abstract

*Providencia* genus is known to harbor certain opportunistic pathogens capable of causing human infections. Here, we report two strains of multidrug-resistant bacteria initially identified as *Providencia rettgeri* by mass spectrometry, but genome analysis revealed their ANI (79.84–84.20%) and dDDH (21.1–25.6%) values to fall below the accepted species threshold for known *Providencia* species. We therefore propose that these isolates be recognized as a novel species, *Providencia xianensis* sp. nov. Alarmingly, both strains, isolated from locations far apart, exhibited resistance to last-resort antibiotics, indicating their possible wide distribution, underscoring the urgency for immediate attention and enhanced surveillance for this emerging multidrug-resistant pathogen.

**Supplementary Information:**

The online version contains supplementary material available at 10.1007/s10096-024-04821-y.

## Introduction

The *Providenci*a genus, nestled within the *Enterobacterales* order as part of the *Proteeae* tribe alongside relatives *Proteus* and *Morganella*, consists of Gram-negative bacteria that are adept at producing urease [1, 2]. The members of *Providencia* thrive in a variety of environments, ranging from aquatic and soil ecosystems to animal hosts [1]. At the time of writing, per the information available in the List of Prokaryotic names with Standing in Nomenclature (https://lpsn.dsmz.de/genus/providencia), the genus includes 14 distinct species, with *Providencia rettgeri*, *Providencia stuartii*, *Providencia alcalifaciens,* and *Providencia hangzhouensis* standing out due to their pronounced relevance to human infections [3–11]. Renowned for their association with urinary tract infections, these pathogens also possess the capability to inflict a broader spectrum of severe ailments, such as ocular diseases [12], peritonitis [13], neonatal septicemia or bloodstream infections [14] and meningitis [15]. Such versatility highlights the genus's extensive pathogenic potential, extending well beyond the confines of urinary tract afflictions.

In 2023, from our routine bacterial collection, we acquired two clinical strains (3007 and 23021821) from two Chinese grade-A tertiary hospitals in Henan and Shaanxi Province, initially labeled as *Providencia*. Routine MALDI-TOF MS identified them as *Providencia rettgeri*. Given the complexities identified in previous species classifications within this genus [3], we performed genomic sequencing on these strains. Genome analysis revealed that they constitute a novel species within the *Providencia* genus, proposed to be named *Providencia xianensis* sp. nov.

## Results

### Strain isolation and characteristics

Strain 3007 was isolated from a patient with proteinuria and septic shock. Strain 23021821 came from a septic shock patient's ascites sample, linked to intra-abdominal infection. The two strains were isolated from patients in two geographically distinct hospitals, located in Henan and Shaanxi provinces, respectively. Moreover, the strains were collected at different time points, with no temporal overlap between the two cases. Both Strain 3007 and Strain 23021821 were identified as *Providencia rettgeri* by MALDI-TOF MS, scoring 9.254 and 9.596 points, respectively. Both strains are resistant to multiple antibiotics: ampicillin, ceftazidime, tetracycline, tigecycline, polymyxin B, colistin, trimethoprim/sulfamethoxazole, and ciprofloxacin. However, they are susceptible to aztreonam (Table 1).

**Table 1:** Antimicrobial susceptibilities of the strain 3007 and 23021821

Antibiotics	3007		23021821	
	MIC (mg/L)	Category^a^	MIC (mg/L)	Category
Ampicillin	> 128	R	64	R
Ceftazidime	128	R	128	R
Cefepime	0.25	S	32	R
Imipenem	8	R	64	R
Meropenem	0.25	S	8	R
Amikacin	2	S	2	S
Aztreonam	2	S	< 0.1	S
Gentamicin	16	R	2	S
Tigecycline	8	R	4	R
Tetracycline	64	R	16	R
Polymyxin B	> 128	R	> 128	R
Colistin	> 128	R	> 128	R
Trimethoprim-sulfamethoxazole	> 128	R	> 128	R
Ciprofloxacin	32	R	16	R

^a^: S, susceptible; R, resistant

The genetic basis for the resistance observed in both strains includes the *tet(59)* gene (tetracycline resistance), the *dfrA1* gene (trimethoprim/sulfamethoxazole resistance), *lnu(F)* (lincosamide resistance), and *arr-3* (rifamycin resistance)(Table S1**)**. Beta-lactam resistance (ampicillin, ceftazidime) is apparent, with Strain 23021821 additionally resistant to cefepime, imipenem, and meropenem due to the presence of *bla*_NDM-1_. Conversely, Strain 3007 exhibits sensitivity to cefepime and meropenem, attributable to its sole possession of the *bla*_DHA-1_ gene, lacking the capacity to hydrolyze carbapenems. Notably, Strain 3007 demonstrated unexpected imipenem resistance, possibly related to *bla*_DHA-1_'s weak false-positive AmpC expression [16]. Additionally, Strain 3007's resistance to gentamicin is associated with the *aac(3)-IVa* gene. Both strains exhibit significant colistin resistance (MIC > 128 mg/L), consistent with previous findings of inherent resistance in this genus [17].

### Phylogeny represents a unique branch in genus *Providencia*

**Fig. 1:**
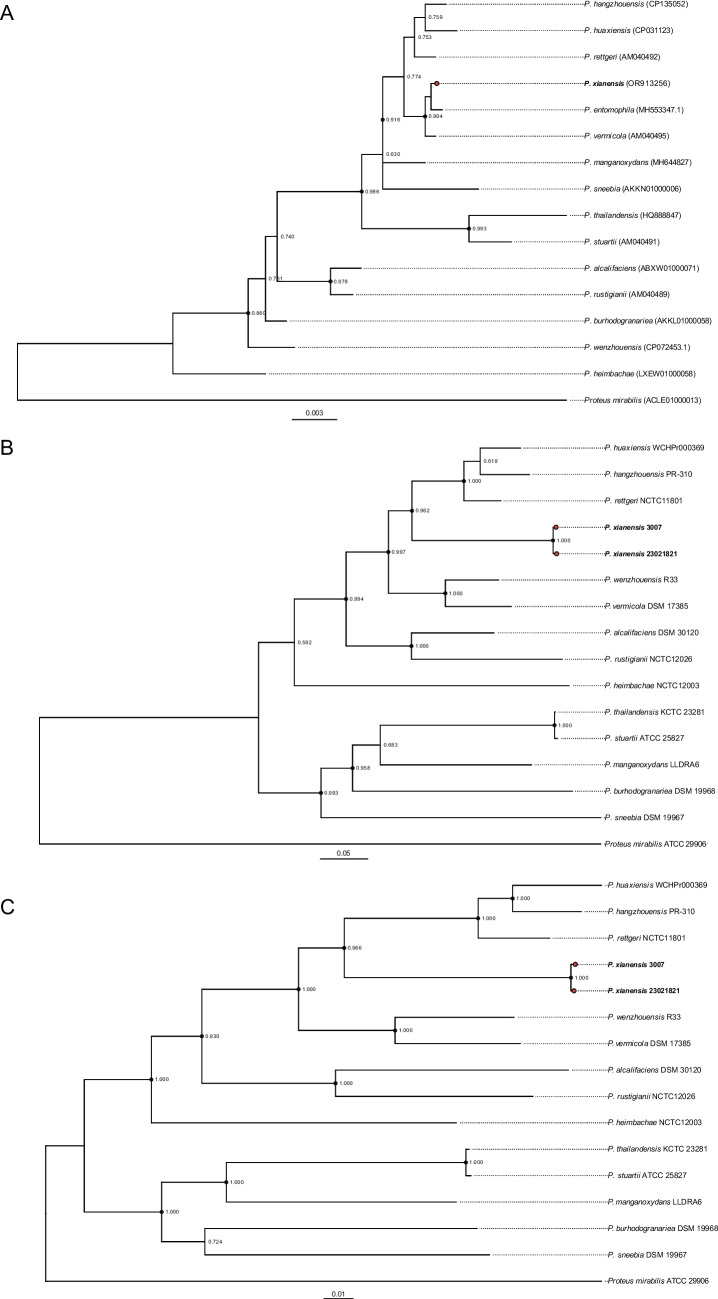
Maximum-likelihood phylogenetic trees of the *Providencia* genus, represented in three parts. **A** depicts the tree constructed from 16S rRNA gene sequences. **B** presents the tree based on concatenated sequences of five housekeeping genes. **C** illustrates the tree derived from whole-genome SNPs. All parts are rooted using *Proteus mirabilis* ATCC 29906 as the outgroup. Branch nodes with bootstrap values above 50%, ascertained from 1,000 resamplings, are marked on each tree. Nodes exceeding 80% bootstrap support are distinctly indicated with black circles. The strains studied are highlighted with red circles

Phylogenetic analysis, including 16S rRNA gene sequencing, multilocus sequence analysis (MLSA) of five housekeeping genes (*fusA*, *gyrB*, *ileS*, *lepA*, and *leuS*), and core single nucleotide polymorphism (SNP) analysis derived from whole genome, consistently supported the classification of Strains 3007 and 23021821 as a novel species within the *Providencia* genus. The 16S rRNA gene sequences of both strains were identical (99.5% identity to *Providencia rettgeri*) and confirmed their placement in the *Providencia* genus (Fig. 1A). The MLSA phylogeny, based on 11,739 bp of concatenated gene sequence alignments, distinctly separated Strains 3007 and 23021821 into a unique evolutionary branch with high bootstrap support (90–100%) (Fig. 1B). The core SNP phylogenetic tree displayed a structure largely congruent with the MLSA tree (Fig. 1C), further reinforcing the novel species status of these strains.

### Genotypic identification

To further validate the taxonomic classification, Strains 23021821 and 3007 were subjected to whole-genome sequencing, resulting in draft genomes of 4.88 Mb and 4.35 Mb with GC contents of 40.3% and 40.2%, respectively. The average nucleotide identity (ANI) values comparing Strain 23021821 with type strains of all known *Providencia* species and Strain 3007 revealed a high similarity to strain 3007 (99.28%) but significantly lower similarities to other *Providencia* species (79.84–84.20%, Table 2). Similarly, the in-silico DNA-DNA hybridization (dDDH) values showed the highest correspondence between strains 23021821 and 3007 (95.1%), with markedly lower values when compared with other *Providencia* species (21.1–25.6%, Table 2). These results strongly suggest that strains 3007 and 23021821 belong to the same, novel species within the *Providencia* genus.

**Table 2:** ANI and dDDH values between strains 23021821 and the type strains of *Providencia* species

Species and/or strain	Assembly accession	23021821		3007	
		ANI (%)	dDDH (%)	ANI (%)	dDDH (%)
*Providencia stuartii* ATCC 25827	GCA_000154865.1	80.23	21.5	80.16	21.3
*Providencia manganoxydans* LLDRA6	GCA_016618195.1	80.27	21.6	80.23	21.8
*Providencia burhodogranariea* DSM 19968	GCA_000314855.2	80.02	21.4	79.98	21.4
*Providencia sneebia* DSM 19967	GCA_000314895.2	79.84	21.1	79.95	21.2
*Providencia hangzhouensis* PR-310	GCA_029193595.2	83.91	25.6	84.01	25.6
*Providencia thailandensis* KCTC 23281	GCA_014652175.1	80.21	21.1	80.24	21.2
*Providencia rettgeri* NCTC11801	GCA_900455085.1	84.19	25.6	84.13	25.5
*Providencia huaxiensis* WCHPr000369	GCA_002843235.3	84.20	25.6	84.15	25.5
*Providencia wenzhouensis* R33	GCA_019343475.1	83.07	24.0	83.04	23.8
*Providencia alcalifaciens* DSM 30120	GCA_000173415.1	80.71	21.2	80.66	21.2
*Providencia heimbachae* ATCC 35613	GCA_001655055.1	81.27	22.0	81.16	22.1
*Providencia vermicola* DSM 17385	GCA_020381325.1	83.26	23.7	83.20	23.7
*Providencia rustigianii* DSM 4541	GCA_000156395.1	80.87	21.3	80.82	21.3
*Providencia zhijiangensis* D4759	GCA_030315915.2	80.78	21.3	80.79	21.5
23021821	GCA_034661195.1	-	-	99.28	95.1
3007	GCA_034661215.1	99.28	95.1	-	-

### Phenotypic characterization

**Fig. 2:**
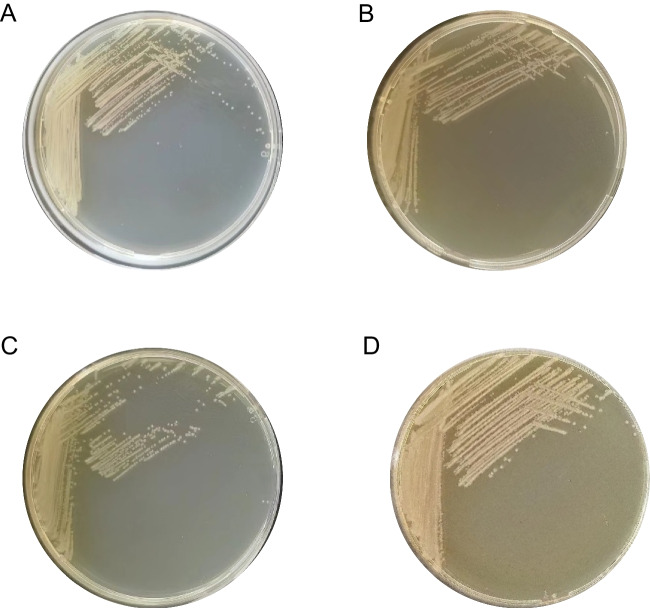
Morphological characteristics of strain 23021821. The morphology of the strain 23021821 grown on different culture media is shown in A-D. **A**: Müller-Hinton agar, **B**: Luria-Bertani agar, **C**: Tryptic soy agar, **D**: Brain Heart Infusion agar. All bacteria were cultured at 37 °C in an aerobic environment

Strain 23021821, chosen as the representative for a detailed description, demonstrated biochemical characteristics consistent with Strain 3007. The biochemical profile of Strain 23021821, in comparison to other known *Providencia* species, is thoroughly detailed in Table 3**.** Microscopic analysis confirmed these bacteria as Gram-negative, motile, and facultatively anaerobic rods. Growth experiments demonstrated Strain 23021821's adaptability to various culture media (TSA, LB, BHI, and MH agar), particularly thriving at 37 °C in aerated conditions. Colony morphology featured circular, raised, yellow, opaque, and smooth textures (Fig. 2). Temperature tolerance ranged from 22–42 °C, peaking at 35–37 °C. The strain tolerated a pH spectrum of 5–10, optimally at 7.0–8.0, and grew in 0–7% NaCl solutions. Additionally, it grew under both aerobic and anaerobic conditions, showing catalase positivity but oxidase negativity.

**Table 3:** Biochemical characteristics of strain 23021821 and type strains of other *Providencia* species^a^

Characteristic	23021821	1	2	3	4	5	6	7	8	9	10	11	12	13	14
API 20E tests:															
β-Galactosidase	-	-	-	-	-	-	-	-	-	-	+	-	-	-	ND
L-Arginine dihydrolase	-	-	-	-	-	-	-	-	-	-	+	-	-	-	ND
Lysine decarboxylase	-	-	-	-	-	-	-	-	-	-	-	-	-	-	-
L-Ornithine decarboxylase	-	-	-	-	-	-	-	-	-	-	+	-	-	-	ND
Citrate utilization	+	+	-	-	+	+	-	-	-	+	+	-	+	+	+
H_2_S production	-	-	-	-	-	-	-	-	-	-	-	-	-	-	-
Urea hydrolysis	+	+	-	-	-	+	+	-	+	-	-	-	+	+	-
Deaminase	+	+	+	+	+	+	+	+	+	+	+	+	+	+	ND
Indole production	-	+	+	+	-	+	+	+	+	+	-	+	+	+	-
Acetoin production	-	-	-	-	-	-	+	-	-	-	+	-	+	-	-
Gelatinase	-	-	-	-	-	-	-	-	-	-	+	-	-	-	ND
D-Glucose	+	+	+	+	+	+	+	+	+	+	+	+	+	+	+
D-Mannitol	+	+	-	+	-	+	-	-	+	-	+	+	+	+	+
Inositol	+	+	-	+	-	+	+	-	-	+	+	+	+	+	-
D-Sorbitol	-	-	-	-	-	-	-	-	+	-	+	-	-	-	ND
L-Rhamnose	-	-	-	-	-	-	+	-	-	-	+	-	+	-	ND
Sucrose	-	+	-	-	-	-	-	-	-	+	+	-	-	-	ND
Melibiose	-	-	-	-	-	-	-	-	-	-	+	-	-	-	ND
Amygdalin	+	-	-	-	-	+	+	-	+	-	-	-	+	+	ND
L-Arabinose	-	-	-	+	-	-	-	-	-	-	+	+	-	-	ND
API 50CHE tests:															
Aesculin	+	-	+	+	+	+	+	+	+	-	+	-	ND	ND	ND
Arbutin	+	-	-	+	+	+	-	-	+	-	+	-	ND	ND	ND
Cellobiose	-	-	-	-	-	-	-	-	-	-	+	-	ND	ND	ND
glycerol	+	+	-	-	-	-	+	-	-	+	+	-	ND	ND	-
2-ketogluconate	-	-	-	-	-	+	-	-	-	-	+	+	ND	ND	ND
D-Lyxose	-	+	-	-	-	-	-	-	-	+	-	-	ND	ND	ND
salicin	+	-	-	+	-	+	-	-	+	-	+	-	ND	ND	+
D-Xylose	-	-	-	-	-	-	-	-	+	-	+	-	ND	ND	ND

^a^ Strains: 1, *P. manganoxydans* LLDRA6; 2, *P. alcalifaciens* DSM 30120; 3, *P. burhodogranariea* DSM 19968; 4, *P. heimbachae* DSM 3591; 5, *P. huaxiensis* KCTC 62577; 6, *P. rettgeri* DSM4542; 7, *P. rustigianii* DSM 4541; 8, *P. sneebia* DSM 19967; 9, *P. stuartii* DSM 4539; 10, *P. thailandensis* KCTC 23281; 11, *P. vermicola* DSM 17385; 12, *P. hangzhouensis* PR-310; 13, *P. entomophila* IO-23; 14, *P. wenzhouensis* R33. Data for species other than *P. xianensis* 23021821 are from reference [3, 6]. ND, not determine

### Description of *Providencia xianensis* sp. nov.

*Providencia xianensis* (xi.an.en'sis. N.L. fem. adj. *xianensis*, of Xi'an, referring to Xi'an City, Shaanxi Province, where the organism was isolated).

The cells are Gram-negative, motile, facultatively anaerobic rods, positive for catalase, and negative for oxidase. They exhibit robust growth on various culture media, including TSA, LB, BHI, and MH agar, especially at an optimal temperature of 37 °C in aerated conditions. The colonies are circular, raised, yellow, opaque, and smooth-textured. Growth is supported in a pH range of 5 to 10, with an optimum at pH 7–8, and in 0–7% (w/v) NaCl concentrations. Biochemical tests indicate positive results for deaminase activity and the fermentation of D-glucose, D-mannitol, inositol, amygdalin, aesculin, arbutin, glycerol, and salicin, as well as for urea hydrolysis and citrate utilization.

The type strain, 23021821^T^ (CCTCC AB 2023264^T^ = NBRC 116615^T^), was isolated from a sample of ascites specimen of Xi'an City, Shaanxi Province, China. It has a DNA G + C content of 40.3%.

## Discussion

This study isolated two multidrug-resistant strains from patients in separate hospitals. Genomic and phylogenetic analyses identified them as a novel *Providencia* species, proposed as *Providencia xianensis* sp. nov. This addition to the genus, which includes known pathogens like *P. rettgeri*, *P. stuartii*, and *P. hangzhouensis*, advances our understanding of *Providencia*'s role in human infections. The discovery of *Providencia xianensis* sp. nov. emphasizes the importance of ongoing clinical awareness in emerging unrecognized new species that are pathogenic to humans.

It is noteworthy that both strains were initially misidentified as *P. rettgeri* using MALDI-TOF MS, a misclassification also observed with *P. hangzhouensis* [3]. Such misidentifications can lead to errors in clinical diagnosis and treatment. To this end, we utilized diverse molecular and biochemical methods to conclusively determine the species status of these strains, affirming their novelty. It is worth noting that the two *Providencia xianensis* strains were isolated in clinical setting in two separate and geographically far apart locations. Given the spatial and temporal separation of the two isolates, there appears to be no epidemiological link or contact between the cases from which these strains were obtained. Further studies are needed to determine the prevalence of such strains clinically.

Furthermore, these strains displayed resistance to critical antibiotics such as tigecycline, colistin, and carbapenem. Colistin and tigecycline resistance appears common in this genus, but the underlying mechanisms are yet to be elucidated [17]. Interestingly, Strain 3007 demonstrated resistance to imipenem, attributed to the presence of DHA-1 beta-lactamase. This discrepancy between phenotype and resistance could result from the induction of the AmpC enzyme by imipenem. Similar reports of *bla*_DHA-1_ encoding AmpC in *Klebsiella pneumoniae* have been reported, albeit with occasional weak false-positive results [16].

However, our study is limited by its small sample size, which may not fully capture the clinical distribution and diversity of this novel species. Future research should expand sample collection and conduct comprehensive epidemiological studies in varied clinical settings. Additionally, in-depth functional studies are necessary to decipher the resistance mechanisms and pathogenicity of this novel species.

In summary, the identification of *Providencia xianensis* sp. nov. enriches our knowledge of the *Providencia* genus and highlights the imperative for accurate detection and monitoring of emerging multidrug-resistant bacteria in clinical settings. Our findings pave the way for further investigations into its role in human disease and the management of antibiotic resistance.

### Supplementary Information

Below is the link to the electronic supplementary material.

Supplementary file1 (DOCX 32 KB)

## Data Availability

For detailed methodological information, please refer to the Supplementary Materials section. The genome sequences of the two isolates from our study are available in the GenBank database under BioProject PRJNA1046670, and their 16S rRNA sequences under accession number OR913256.
