# Supplementary material for: Novel Providencia xianensis sp. nov.: A multidrug-resistant species identified in clinical infections
Source: Eur J Clin Microbiol Infect Dis. 2024 May 7;43(7):1461–7. doi: 10.1007/s10096-024-04821-y (PMC11271419; doi:10.1007/s10096-024-04821-y)
Supplement: Supplementary file 1 — Supplementary file1 (DOCX 32 KB) [file 10096_2024_4821_MOESM1_ESM.docx]

Supplementary Materials for

**Novel** ***Providencia xianensis* sp. nov.: A Multidrug-Resistant Species Identified in Clinical Infections**

Xu Dong *et al.*

Corresponding author: Ying Zhang, yzhang207@zju.edu.cn

**The file includes:**

Materials and Methods

Table S1

References

**Materials and Methods**

**Strains and species identification**

In 2023, during our collection of bacterial strains from two grade-A tertiary hospitals, each with over 3,000 beds, one in Henan and the other in Shaanxi province, we encountered two highly drug-resistant strains isolated at different times. Primary species identification of these strains was conducted using MALDI-TOF mass spectrometry (MS) (Autof MS1000, Autobio Diagnostics, China), and Autof Acquirer (Version V2-V2.0.196) containing 5189 microbial species from 17800 microbial strains was used to analyze the results, followed by whole genome sequencing (see below).

**Antibiotic susceptibility testing**

Minimum inhibitory concentrations (MICs) were determined by the agar dilution method, according to the Clinical and Laboratory Standards Institute (CLSI) guidelines [1]. The breakpoints for *Enterobacterales* specified in CLSI M100-ED33 were employed for interpretation, except for tigecycline, where the European Committee on Antimicrobial Susceptibility Testing (EUCAST; http://www.eucast.org/) guidelines were utilized due to the absence of CLSI breakpoints.

**Whole genome sequencing and analysis**

Genomic DNA from the two strains was extracted using the QIAamp DNA minikit (Qiagen, Hilden, Germany), followed by whole genome sequencing with the Illumina HiSeq 2500 platform. The raw sequencing reads were refined using fastp v0.23.2 [2], and the trimmed paired-end reads were then assembled de novo with shovill v1.1.0 (https://github.com/tseemann/shovill), discarding contigs shorter than 200 bp. Average nucleotide identity (ANI) and in silico DNA-DNA hybridization (dDDH) values were calculated using fastANI v1.32 [3] and the genome-to-genome distance calculator (formula 2) [4], respectively. A cutoff of ≥96% ANI [5] or ≥70.0% dDDH [4] values was used to define a bacterial species. Antimicrobial resistance genes were identified within these genome sequences through ABRicate (<https://github.com/tseemann/abricate>). The ORF prediction and gene function annotation of the genomes were performed using Bakta v1.8 [6].

**Phylogenetic analysis**

The 16S rRNA gene sequences from the two strains were amplified by PCR using universal primers 27F and 1492R [7]. Corresponding gene sequences from other species were retrieved from the EzBioCloud database [8]. These sequences were aligned using MAFFT v7.505 [9] and refined with trimal v1.4 [10]. For a multilocus sequence analysis, the complete sequences of five housekeeping genes encoding translational elongation factor EF-G (*fusA*), DNA gyrase subunit B (*gyrB*), isoleucyl-tRNA synthetase (*ileS*), translation elongation factor EF-4 (*lepA*) and leucyl-tRNA synthetase (*leuS*) of the two strains, as well as those from other available type strain genomes, were extracted, concatenated, and then compared utilizing MAFFT and trimal [11]. Whole genome SNPs for each isolate were identified using Snippy (https://github.com/tseemann/snippy). Phylogenetic trees, were constructed with FastTree [12], employing the maximum likelihood method, and were visualized using ggtree [13].

**Phenotypic characterization**

As described previously [14], the Gram staining and biochemical characterization of strain 23021821 were performed using bioMérieux API 20E and API 50CH kits, following the manufacturer’s guidelines. Oxidase activity was assessed with bioMérieux oxidase reagent. Cell morphology was observed under an optical microscope after a 16-hour incubation on LB agar at 37°C. Growth patterns were analyzed on various agar media, including tryptic soy agar (TSA), Luria-Bertani (LB) agar, brain heart infusion (BHI) agar, and Müller-Hinton (MH) agar. To determine optimal growth conditions, we incubated the cells in tryptic soy broth (TSB) for 24 hours at temperatures ranging from 4 to 44°C, pH levels from 4.0 to 11.0, and salt concentrations between 0 and 9% (w/v) in a thermostatic incubator. Anaerobic growth was tested by incubating cells on BHI agar in an anaerobic bag for 48 hours. Catalase activity was determined by the formation of bubbles upon the addition of 3% (v/v) H_2_O_2_ to fresh biomass grown on LB agar for 24 hours.

**Table S1** Antibiotic resistance genes carried by the two strains.

| **Antibiotic type** | **23021821** | **3007** |
| --- | --- | --- |
| Aminoglycoside | *aadA1* | *aac(6')-Ib-cr5* |
|  |  | *aph(4)-Ia* |
|  |  | *aac(3)-IVa* |
| β-lactam | *bla*_NDM-1_ | *bla*_DHA-1_ |
| Rifamycin | *arr-3* | *arr-3* |
| Tetracycline | *tet(59)* | *tet(59)* |
| Quinolone | *qnrD1* | *qnrD1* |
| Trimethoprim | *dfrA1* | *dfrA1* |
| Lincosamide | *lnu(F)* | *lnu(F)* |
| Bleomycin | *ble-MBL* |  |
| Sulfonamide |  | *sul1* |
|  |  | *sul2* |
| Macrolide |  | *mph(E)*  *msr(E)* |

**Reference**

[1] Humphries R, Bobenchik AM, Hindler JA, Schuetz AN (2021) Overview of Changes to the Clinical and Laboratory Standards Institute Performance Standards for Antimicrobial Susceptibility Testing, M100, 31st Edition. J Clin Microbiol 59 (12):e0021321

[2] Chen S, Zhou Y, Chen Y, Gu J (2018) fastp: an ultra-fast all-in-one FASTQ preprocessor. Bioinformatics 34 (17):i884-i890

[3] Croucher NJ, Page AJ, Connor TR, Delaney AJ, Keane JA, Bentley SD, Parkhill J, Harris SR (2015) Rapid phylogenetic analysis of large samples of recombinant bacterial whole genome sequences using Gubbins. Nucleic Acids Res 43 (3):e15

[4] Meier-Kolthoff JP, Auch AF, Klenk HP, Goker M (2013) Genome sequence-based species delimitation with confidence intervals and improved distance functions. BMC Bioinformatics 14:60

[5] STACKEBRANDT E, GOEBEL BM (1994) Taxonomic Note: A Place for DNA-DNA Reassociation and 16S rRNA Sequence Analysis in the Present Species Definition in Bacteriology. International Journal of Systematic and Evolutionary Microbiology 44 (4):846-849

[6] Schwengers O, Jelonek L, Dieckmann MA, Beyvers S, Blom J, Goesmann A (2021) Bakta: rapid and standardized annotation of bacterial genomes via alignment-free sequence identification. Microb Genom 7 (11)

[7] Weisburg WG, Barns SM, Pelletier DA, Lane DJ (1991) 16S ribosomal DNA amplification for phylogenetic study. J Bacteriol 173 (2):697-703

[8] Yoon SH, Ha SM, Kwon S, Lim J, Kim Y, Seo H, Chun J (2017) Introducing EzBioCloud: a taxonomically united database of 16S rRNA gene sequences and whole-genome assemblies. Int J Syst Evol Microbiol 67 (5):1613-1617

[9] Katoh K, Misawa K, Kuma K, Miyata T (2002) MAFFT: a novel method for rapid multiple sequence alignment based on fast Fourier transform. Nucleic Acids Res 30 (14):3059-3066

[10] Capella-Gutierrez S, Silla-Martinez JM, Gabaldon T (2009) trimAl: a tool for automated alignment trimming in large-scale phylogenetic analyses. Bioinformatics 25 (15):1972-1973

[11] Li Z, Liao F, Ding Z, Chen S, Li D (2022) Providencia manganoxydans sp. nov., a Mn(II)-oxidizing bacterium isolated from heavy metal contaminated soils in Hunan Province, China. Int J Syst Evol Microbiol 72 (8)

[12] Price MN, Dehal PS, Arkin AP (2009) FastTree: computing large minimum evolution trees with profiles instead of a distance matrix. Mol Biol Evol 26 (7):1641-1650

[13] Yu G, Smith, David K., Zhu, Huachen, Guan, Yi, Lam, Tommy Tsan-Yuk (2017) ggtree: an r package for visualization and annotation of phylogenetic trees with their covariates and other associated data. Methods in Ecology and Evolution 8

[14] Wu W, Feng Y, Zong Z (2020) Precise Species Identification for Enterobacter: a Genome Sequence-Based Study with Reporting of Two Novel Species, Enterobacter quasiroggenkampii sp. nov. and Enterobacter quasimori sp. nov. mSystems 5 (4)
